# Decoding tumor stage by peritumoral and intratumoral radiomics in resectable esophageal squamous cell carcinoma

**DOI:** 10.1007/s00261-023-04061-2

**Published:** 2023-10-13

**Authors:** Xian-Zheng Tan, Rong Ma, Peng Liu, Chang-Hui Xiao, Hui-Hui Zhang, Fan Yang, Chang-Hong Liang, Zai-Yi Liu

**Affiliations:** 1 Department of Radiology, Hunan Provincial People’s Hospital (The First Affiliated Hospital of Hunan Normal University), Changsha, 410005 Hunan China; 2 Department of Radiology, The First People’s Hospital of Changde City, Changde, 415000 Hunan China; 3 Department of Radiology, Guangdong Provincial People’s Hospital (Guangdong Academy of Medical Sciences), Southern Medical University, Guangzhou, 510180 Guangdong China; 4 Guangdong Provincial Key Laboratory of Artificial Intelligence in Medical Image Analysis and Application, Guangzhou, 510180 Guangdong China

**Keywords:** Esophageal squamous cell carcinoma, Tumor stage, Computed tomography, Radiomics, Prognosis

## Abstract

**Purpose:**

To evaluate the potential application of radiomics in predicting Tumor-Node-Metastasis (TNM) stage in patients with resectable esophageal squamous cell carcinoma (ESCC).

**Methods:**

This retrospective study included 122 consecutive patients (mean age, 57 years; 27 women). Corresponding tumor of interest was identified on axial arterial-phase CT images with manual annotation. Radiomics features were extracted from intra- and peritumoral regions. Features were pruned to train LASSO regression model with 93 patients to construct a radiomics signature, whose performance was validated in a test set of 29 patients. Prognostic value of radiomics-predicted TNM stage was estimated by survival analysis in the entire cohort.

**Results:**

The radiomics signature incorporating one intratumoral and four peritumoral features was significantly associated with TNM stage. This signature discriminated tumor stage with an area under curve (AUC) of 0.823 in the training set, with similar performance in the test set (AUC 0.813). Recurrence-free survival (RFS) was significantly different between different radiomics-predicted TNM stage groups (Low-risk vs high-risk, log-rank *P* = 0.004). Univariate and multivariate Cox regression analyses revealed that radiomics-predicted TNM stage was an independent preoperative factor for RFS.

**Conclusions:**

The proposed radiomics signature combing intratumoral and peritumoral features was predictive of TNM stage and associated with prognostication in ESCC.

**Graphical abstract:**

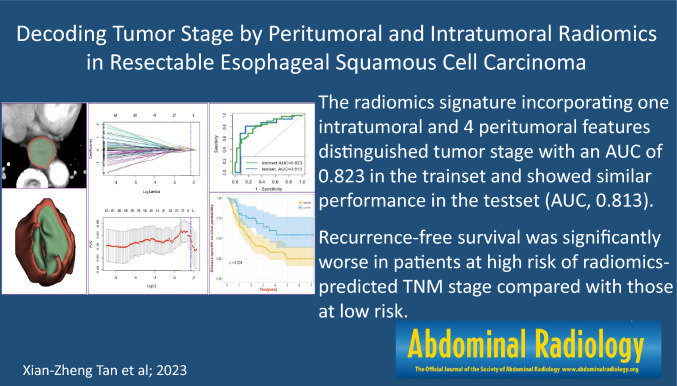

**Supplementary Information:**

The online version contains supplementary material available at 10.1007/s00261-023-04061-2.

## Introduction

Esophageal cancer remains an important cancer worldwide and is responsible for over 600,000 new cases in 2020 and an estimated 544,000 deaths, ranking seventh for incidence and sixth for mortality globally [1]. Esophageal squamous cell cancer (ESCC), as the dominant histological subtype, comprises over 90% of all esophageal cancer cases in the high-risk areas such as East and Central Asia [2, 3].

Local-regional staging is essential for decision making and prognostication of esophageal carcinoma [1, 4]. Important findings were, first, that staging accuracy of current imaging modalities is still relatively inadequate [5–7]. Second, clinical staging, currently based largely on imaging, cannot predict the survival as accurately as pathologic staging, which overestimated the survival of early-stage tumor and underrated the survival of advanced-stage tumor [8]. Therefore, new tools for accurate clinical staging have to be developed to facilitate precision cancer care.

Radiomics enable non-invasive decoding of clinical staging in various cancers including ESCC [9–11]. However, previous radiomics studies mainly focused on the intratumoral region alone, whereas there is paucity of data evaluating the potential value of peritumoral radiomics features for predicting ESCC staging. Recently, peritumoral radiomics features have been shown to be predictive in treatment response assessment in ESCC [12]. Therefore, the primary objective of the study was to assess the ability of intratumoral and peritumoral radiomics in predicting local-regional staging in ESCC. The secondary objectives were to investigate associations between radiomics-based staging phenotype and patient survival.

## Materials and methods

This retrospective study was approved by the ethics committee of our institution (no. ky-2023-109) without the requirement of written informed consent.

### Patients

**Fig. 1:**
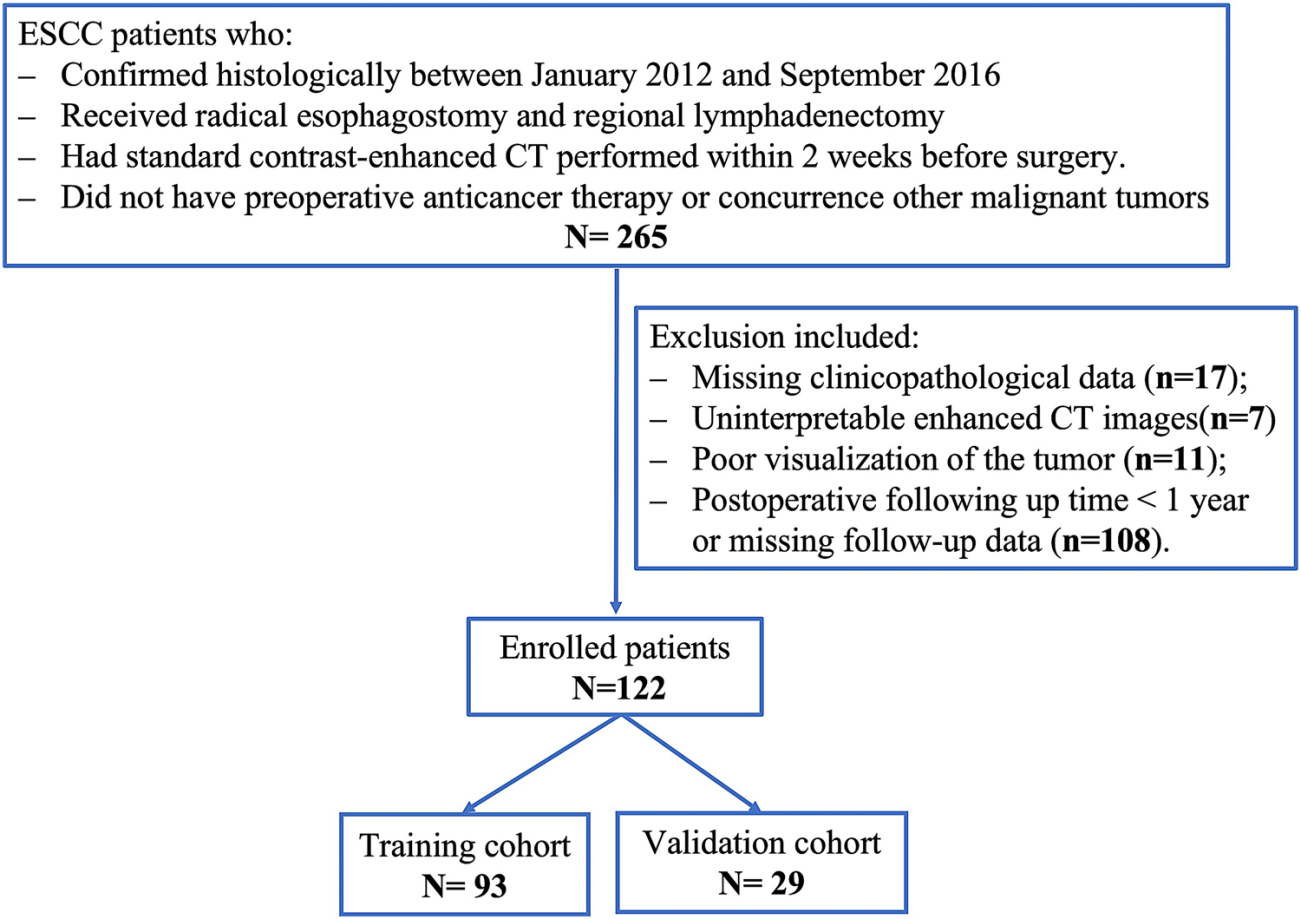
Flow diagram of patient enrollment, eligibility, and exclusion criteria. *ESCC* esophageal squamous cell carcinoma, *CT* computed tomography

Esophageal cancer patients who underwent radical esophagostomy and regional lymphadenectomy (i.e., pathological T1-4aN1-2M0) in our institution from January 2012 to September 2016 were retrospectively recruited. Inclusion criteria were as follows: (1) ESCC confirmed histologically and (2) standard contrast-enhanced computed tomography (CT) performed within 2 weeks before surgery. Exclusion criteria included (1) preoperative anticancer therapy; (2) concurrence other malignant tumors; (3) missing clinicopathological data (preoperative blood-routine characteristics, pathological data for definite TNM stage, etc.); (4) uninterpretable enhanced CT images; and (5) Postoperative following up time < 1 year. One hundred twenty-two patients who met the criteria were allocated randomly to the training cohort (*n* = 93) and internal validation cohort (*n* = 29) in a 3:1 ratio. Patient enrollment pathway is shown in Fig. 1. These patients were previously reported as part of a radiomics study [13]. Yet, the current study exploits a different study purpose, methodology, and results as compared with the prior publication. Whereas previous study dealt with intratumoral radiomics and focused on prediction of lymph node (LN) metastasis, the present study evaluates the diagnostic performance of intratumoral and peritumoral radiomics in predicting local-regional staging and prognostic value of radiomics-predicted Tumor-Node-Metastasis (TNM) stage.

### Clinicopathological characteristics

Clinical information, including demographic data (age, sex), laboratory test (serum albumin, fibrinogen, blood-routine characteristics), and histopathological reports (tumor site, grade, Tumor stage, Node stage) were collected from electronic medical record databases. Neutrophil-to-lymphocyte ratio (NLR) and platelet-to-lymphocyte ratio (PLR) were calculated based on neutrophil, lymphocyte, and platelet count within 2 weeks before surgery. The threshold values for serum albumin and fibrinogen used here were 35 g/L and 4 g/dL, respectively. The pathologic stage was defined according to the Union for International Cancer Control TNM staging system (8th edition) [8, 14]. Stage I and II were classified as the early-stage, and stage III and IV the late-stage.

### Follow‑up strategy

Patients were followed up every 3 months for the 1st year after surgery, every 6 months for thereafter. At each outpatient visit, thoraco-abdominal CT scans, brain magnetic resonance images or brain CT scans and bone scans were routinely performed to detect any evidence of recurrence. The recurrence date was recorded as the date when the aforementioned scans first showed signs of recurrence. Recurrence-free survival (RFS) was defined as the duration from the date of surgery to the first radiographic detection of recurrence, death, or the last follow-up was set as the end point.

### CT acquisition

All patients underwent contrast-enhanced chest CT using a 64-slice LightSpeed VCT (GE Healthcare), which was performed in the axial plane with 5-mm-thick sections.

Details on the imaging protocols are shown in Supplementary S1 (online). Arterial-phase CT images, as the optimal one for visualization of esophageal cancer, were retrieved from picture archiving and communication system (Carestream, Canada) for tumor annotation [15]. CT-reported lymph node (LN) status was assessed in consensus on the pretreatment CT by two radiologists (Z.T. and R.M., with 12 and 6 years of clinical experience in esophageal imaging, respectively). LN short-axis diameter greater than 10 mm was defined as a radiological positive nodal status [16].

### Tumor segmentation and feature extraction

**Fig. 2:**
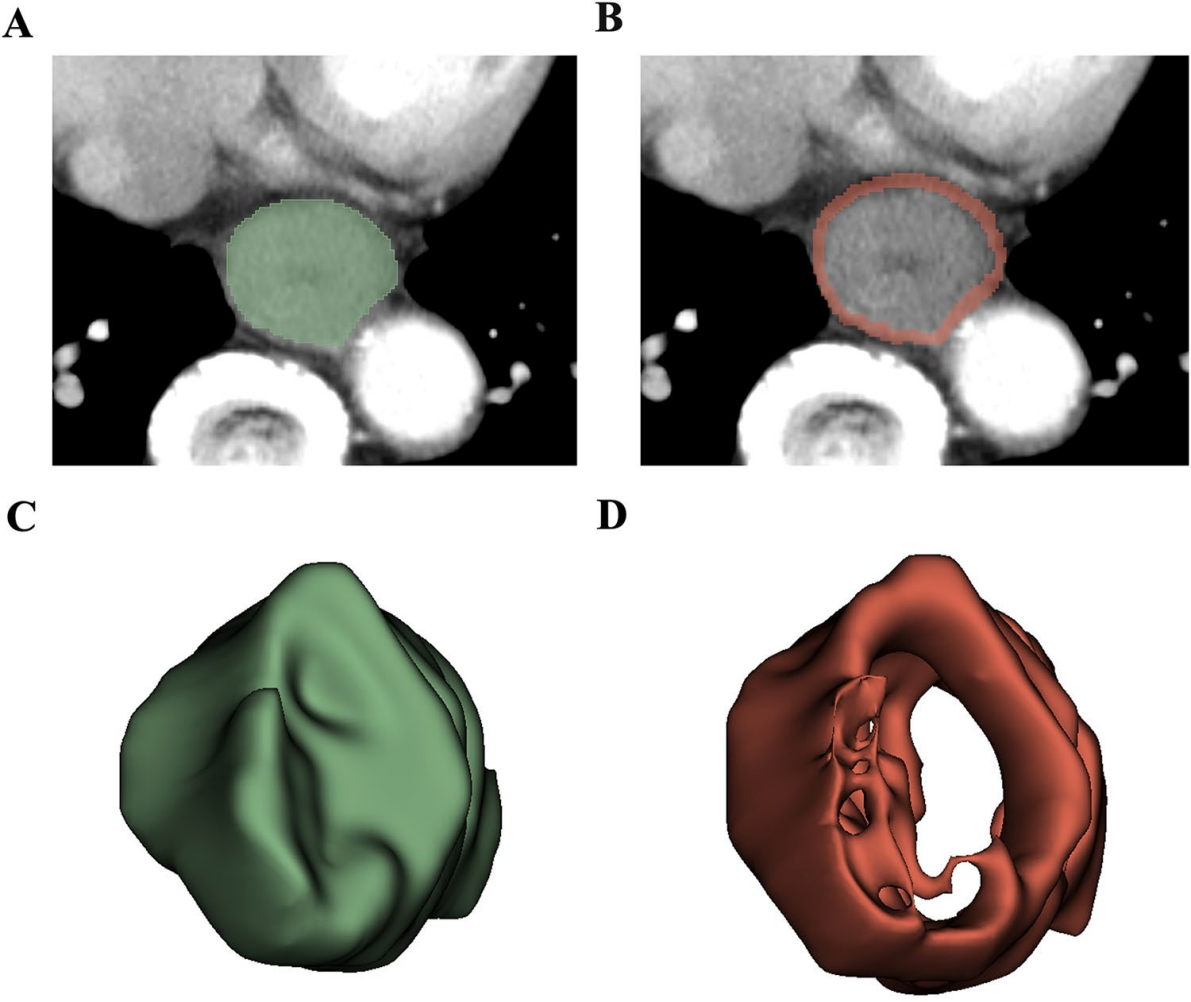
Lesion segmentation for radiomics analysis. First, **A** Region of interest was manually segmented in axial view to obtain intratumoral mask, **B** then the peritumoral masks with a radial distance of 3 mm were semiautomatically generated using morphologic outward dilation by 2 mm and inside erosion by 1 mm of the tumor boundaries, with airway, lung, left atrium, aorta, vertebrae, and azygos excluded manually. **C**, **D** Three-dimensional view of the intratumoral and peritumoral volumes of interest

Tumor segmentation and radiomic features extraction were performed by using the open software 3D Slicer (version 4.10.2, http://www.slicer.org). The intratumoral three-dimensional regions of interests (ROIs) covering the whole tumor in all patients were manually delineated slice by slice on the CT images by the one investigator (R.M), who was blinded to pathological TNM stage. After intratumoral segmentation, peritumoral masks with a radial distance of 3 mm were automatically created using morphologic outward dilation by 2 mm and inside erosion by 1 mm of the tumor boundaries. Airway, lung, left atrium, aorta, vertebrae, and azygos were manually excluded (Fig. 2). To evaluate the interreader agreement, an independent investigator (X.T) also placed three-dimensional ROIs of the intratumoral and peritumoral areas in a randomly selected subset of 30 patients.

Before features extraction, image normalization was performed by remapping the histogram to fit within μ ± 3σ (μ: mean gray level within the VOI; σ: gray level standard deviation). For each ROI, 1223 quantitative features were extracted, including 14 shape features, 234 first-order features, and 975 second-order texture features derived from gray level dependence matrix (GLDM), gray level co-occurrence matrix (GLCM), gray level run length matrix (GLRLM), gray level size zone matrix (GLSZM), and neighboring gray tone difference matrix (NGTDM).

### Construction of a TNM-related radiomic signature

The reproducibility of features was calculated by using intra-class correlation coefficients (ICC). After being normalized using the *z* score standardization method, all radiomics features were filtered using the criteria of ICC ≥ 0.80 and correlation coefficient ≥ 0.90. The remaining features were then input into the least absolute shrinkage and selection operator (LASSO) logistic regression model to avoid overfitting and construct radiomic signature. The output of LASSO model was converted into a probability score, namely the radiomic score, indicating the individual relative risk for high pathologic tumor stage.

### Statistical analysis

All statistical analyses were performed using R version 4.2.1 (The R Foundation). A two-tailed *P* value less than 0.05 was considered as statistical significance. The clinicopathological characteristics between two datasets were compared with Chi-Square, *t* test or Mann–Whitney *U* test, where appropriate. The discrimination performance of the radiomic signature was quantified by the area under curve (AUC) value in the primary training set and internally validated in the independent test set. To explore the prognostic value of radiomics-predicted TNM stage, the optimum threshold of the radiomic score was determined using the surv_cutpoint function of survival R package. Accordingly, the patients were divided into low- and high-risk groups in the entire cohort, for which the survival outcomes were compared with Kaplan–Meier analysis and the 2-sided log-rank tests. Univariable and multivariable Cox regression analyses were conducted to analyze the relationship between radiomics-predicted TNM stage and RFS.

## Results

### Patient characteristics

After applying the exclusion criteria, 122 patients (mean age, 57 years; 27 women) with ESCC were included for radiomics signature training, internal validation, and survival comparison analysis. The details of clinical-pathologic characteristics are shown in Table 1. High TNM stage in training set and test set was 55.2% and 54.8%, respectively. The median follow-up time was 47.6 months (interquartile range 39.8-63.2). The median RFS duration for the entire cohort was 21.9 months, with 67 of 122 (54.9%) patients experiencing recurrence after complete surgical resection (median RFS time: 19.2 and 22.1 months for training set and test set, respectively). No significant differences in clinicopathological characteristics between two datasets justified their use as training and test sets (see Table 1).

**Table 1:** Baseline characteristics of patients in training and test sets

Characteristics	Trainset (*n* = 93)	Testset (*n* = 29)	*P* value
Age, mean ± SD, years	57.01 ± 8.84	59 ± 8.51	0.288
Gender, no (%)			0.638
Male	71 (76.3%)	24 (82.8%)	
Female	22 (23.7%)	5 (7.2%)	
Tumor location, no (%)			0.896
Upper third	14 (15.1%)	4 (13.8%)	
Middle third	45 (48.4%)	13 (44.8%)	
Lower third	34 (36.6%)	12 (42.4%)	
NLR, median (interquartile range)	2.000 (1.500–3.000)	2.000 (1.500–3.000)	0.524
PLR, median (interquartile range)	121.00 (96.00–171.50)	119.75 (93.5–173.00)	0.904
Fibrinogen			0.645
≤ 4 g/dL	17 (58.6%)	61 (65.6%)	
> 4 g/dL	12 (41.4%)	32 (34.4%)	
Serum albumin			1.000
≤ 35 g/L	7 (24.1%)	22 (23.7%)	
> 35 g/L	22 (75.9%)	71 (76.3%)	
Tumor volume (mm^3^) (interquartile range)	17.92 (12.11, 23.22)	13.78 (7.71, 23.02)	0.175
CT-reported LN status, no (%)			0.169
LN-negative	98 (63.6%)	56 (73.7%)	
LN-positive	56 (36.4%)	20 (26.3%)	
Histological grade			0.474
G1	2 (6.9%)	14 (15.1%)	
G2	20 (69.0%)	55 (59.1%)	
G3	7 (24.1)	24 (25.8%)	
TNM stage (8th)			1.000
Low stage (I–II)	13 (44.8%)	42 (45.2%)	
High stage (III–IVa)	16 (55.2%)	51 (54.8%)	
Recurrence, no (%)			0.806
Absence	12 (41.4%)	43 (46.2%)	
Presence	17 (58.6%)	50 (53.8%)	
Recurrence-free time, months	19.2 (8.2, 40.9)	22.1 (12.2, 42.7)	0.459

*P* value is derived from Chi-Square, *t* test or Mann–Whitney *U* test, where appropriate

*SD* standard deviation, *NLR* neutrophil-to-lymphocyte ratio, *PLR* platelet count to lymphocyte ratio, *CT* computed tomography, *LN* lymph node, *G* grade, *TNM* tumor-node-metastasis

### Radiomics features selection and signature construction

**Fig. 3:**
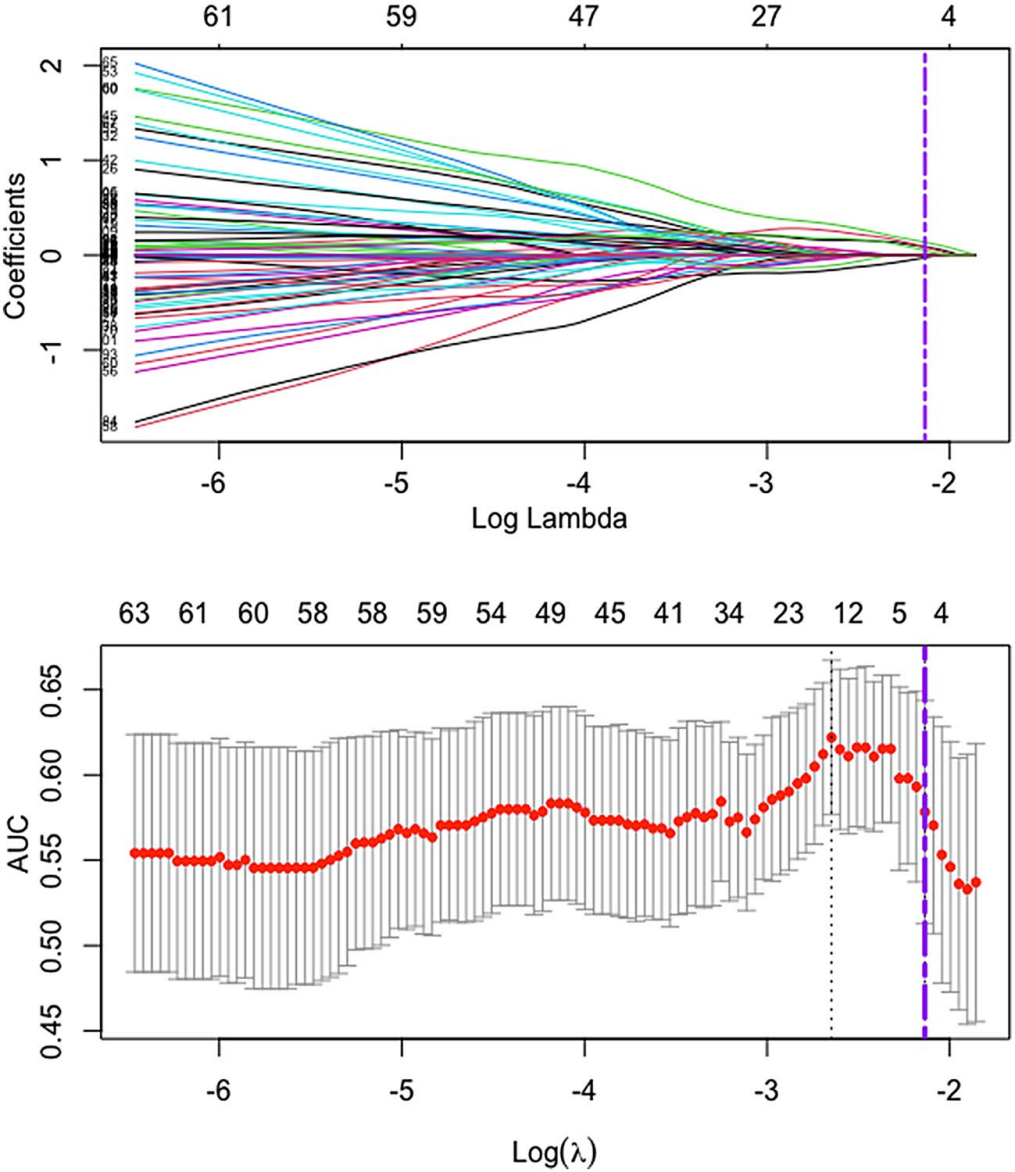
Selection of TNM-associated radiomics features via least absolute shrinkage and selection operator algorithm. Top figure shows the coefficient profiles of 122 radiomics features against the log (*λ*), bottom figure the cross-validation curve. Pink dotted vertical lines were drawn at the optimal value by using fivefold cross-validation and the 1 standard error of the minimum criteria. Five nonzero coefficients were selected. *TNM* tumor-node-metastasis

A total of 2446 features were extracted from two ROIs per patient. After the reproducibility and correlation analysis, a list of 122 features was retained with 2324 ineligible features excluded. Next, five radiomics features, including one intratumoral feature and four peritumoral features, were selected by the LASSO algorithm (Fig. 3) and quantitatively integrated into the radiomics signature. The radiomic score was computed as follows: radiomic score = *0.22885113 − (0.13596893 × Intratumoral_wavelet-LHH.glcm.DifferenceAverage) − (0.02591146 × Peritumoral_log-sigma-1-5-mm-3D.gldm.SmallDependenceLowGrayLevelEmphasis) + (0.08734137 × Peritumoral_wavelet-HLH.firstorderKurtosis) + (0.08575472 × Peritumoral_wavelet-HHH.glszm.SizeZoneNonUniformity) + (0.05001215 × Peritumoral_original.glcmInverseVariance)*.

### Validating the radiomics signature

**Fig. 4:**
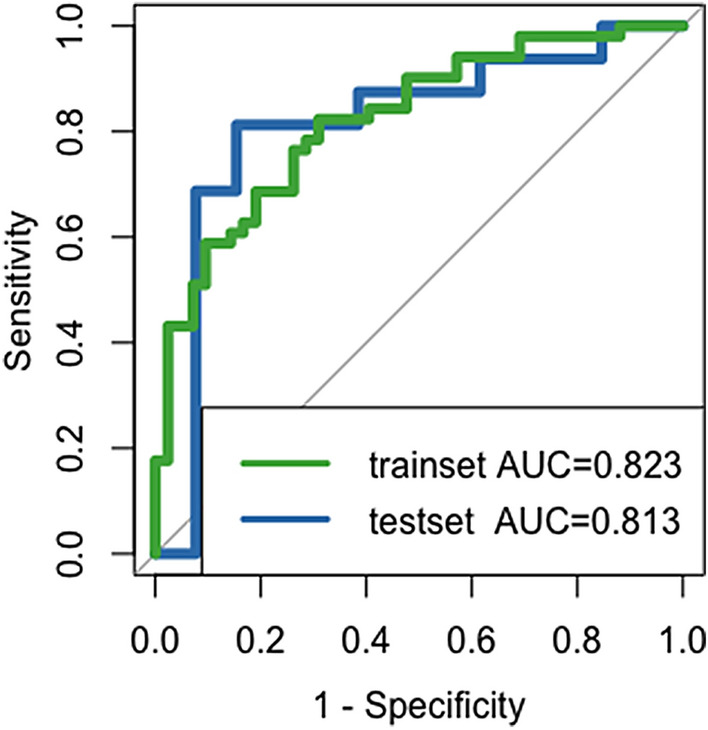
Receiver operating characteristic curves to predict TNM stage for the radiomics signature in the training and test sets. The radiomics signature incorporating one intratumoral and four peritumoral features distinguished tumor stage with an area under curve (AUC) of 0.823 in the trainset, with similar performance in the testset (AUC 0.813)

The receiver operating characteristic curves analysis indicated that the radiomics signature exhibited AUCs of 0.823 (95% CI 0.739–0.906) in the training set, and 0.813 (95% CI 0.632–0.993) in the test set (Fig. 4).

### Survival risk stratification based on the radiomics signature

**Fig. 5:**
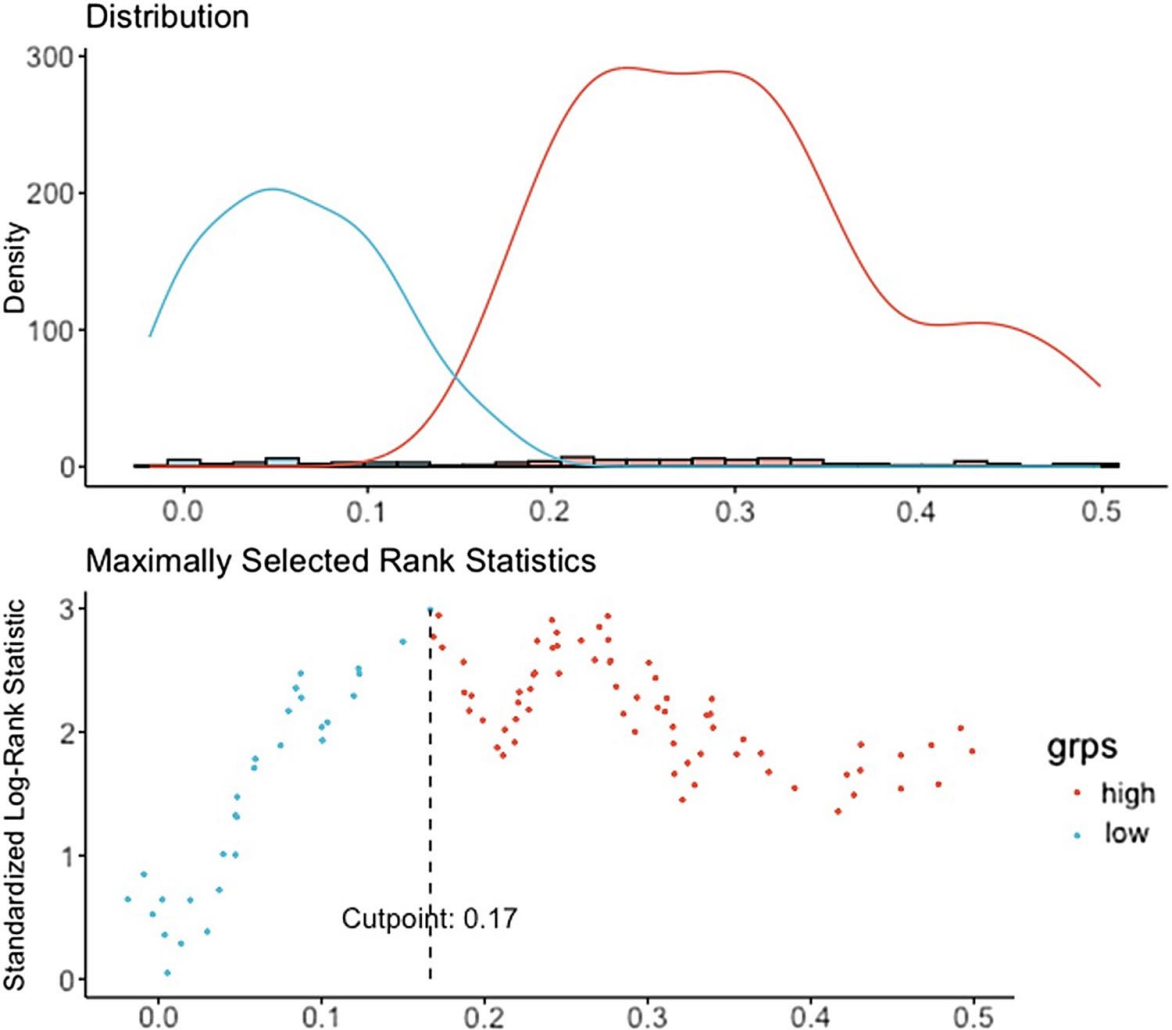
The classification of radiomics-predicted TNM stage status was derived using the optimal threshold of radiomic score (0.17) determined by the surv_cutpoint function of the R package survminer

**Fig. 6:**
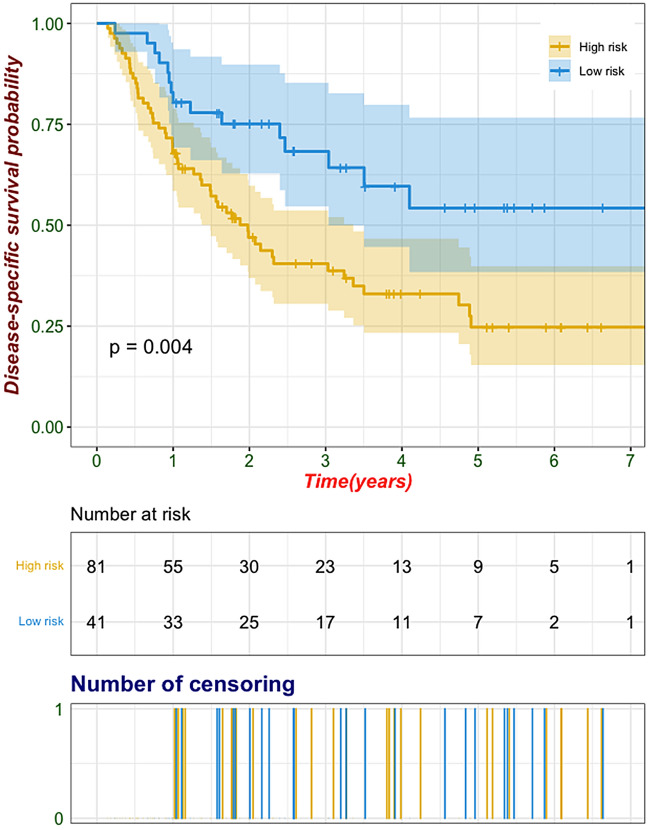
Kaplan–Meier survival analyses stratified by radiomics-predicted TNM stage in the entire cohort (*n* = 122). Recurrence-free survival was significantly worse in patients at high risk compared with those at low risk. Shaded areas represent 95% confidence intervals

To explore the relationship between radiomics-predicted TNM stage and RFS, the entire cohort was clustered into low-risk (radiomic score < 0.17) and high-risk (radiomic score ≥ 0.17) groups according to the optimal cutoff value of the radiomic score determined by the surv_cutpoint function of survival R package (Fig. 5). The distribution of the radiomic score indicated that patients with low scores were commonly associated with favorable RFS, while those with high scores showed an increasing frequency of recurrence (log-rank *P* = 0.004, Fig. 6), with a hazard ratio of 0.47 (95% CI 0.27–0.83, *P* = 0.009). Multivariable Cox regression analysis revealed that radiomics-predicted TNM stage was an independent preoperative factor for RFS (hazard ratio, 0.32 [95% CI 0.17, 0.64]; *P* = 007) following curative-intent resection of ESCC, as was CT-reported LN status (hazard ratio, 1.95 [95% CI 1.13, 3.35]; *P* = 016) (Table 2).

**Table 2:** Uni- and multivariable cox regression analysis of predictors of recurrence-free survival

Variable	Disease-specific survival			
	Univariable analysis		Multivariable analysis	
	Hazard ratio	*P* value	Hazard ratio	*P* value
Age	0.99 (0.97–1.02)	0.645	NA	NA
Sex, men vs women	1.19 (0.65–2.18)	0.578	NA	NA
NLR	1.01 (0.87–1.18)	0.875	NA	NA
PLR	1.00 (1.00–1.00)	0.998	NA	NA
Tumor volume (mm^3^)	1.00 (1.00–1.00)	0.340	NA	NA
Fibrinogen, > 4 g/dL vs ≤ 4 g/dL	1.11 (0.68–1.83)	0.672	NA	NA
Serum albumin, > 35 g/L vs ≤ 35 g/dL	0.78 (0.45–1.38)	0.395	NA	NA
Tumor location				
Upper third vs lower third	0.78 (0.36–1.65)	0.511	NA	NA
Middle third vs lower third	0.72 (0.43–1.20)	0.205	NA	NA
Histological grade				
G2 vs G1	1.63 (0.69–3.84)	0.263	NA	NA
G3 vs G1	1.95 (0.78–4.89)	0.154	NA	NA
CT-reported LN status, positive vs negative	1.82 (1.13–2.95)	0.015	1.95 (1.13–3.35)	0.016
Radiomics-predicted TNM stage, high risk vs low risk	0.47 (0.27–0.83)	0.009	0.32 (0.17–0.64)	0.001

*NLR* neutrophil-to-lymphocyte ratio, *PLR* platelet count to lymphocyte ratio, *G* grade, *CT* computed tomography, *LN* lymph node, *TNM* tumor-node-metastasis

## Discussion

In this study, we developed and validated a radiomics signature for the preoperative prediction of TNM stage in patients with resectable ESCC. The radiomics signature, which combined one intratumoral feature and four peritumoral features, performed well in distinguishing TNM stage with an AUC of 0.823 in the training set, and showed similar discrimination on internal validation (AUC 0.813). The comparable performance implied that the radiomics signature was a robust imaging biomarker in predicting tumor stage. In addition, the staging phenotype predicted by radiomics emerged as an independent preoperative predictor of RFS, thereby providing potentially prognostic information for medical decision making. To our knowledge, this study is the first exploring the combined predictive value of intratumoral and peritumoral radiomics features for tumor stage in ESCC patients, and also the first investigating the prognostic value of radiomics-based staging phenotype.

Pretreatment prediction of TNM stage is important for risk stratification and individualized therapy [4, 17]. Patients with late-stage ESCC are likely to be offered neoadjuvant chemoradiation in hopes of improving survival. Yet, clinical staging based chiefly on imaging modalities is still relatively inaccurate and more precise clinical staging tools are needed [8]. Prior studies supported the potential use of radiomics as a useful tool for tumor stage prediction in different clinical settings [9, 11, 18]. Wu et al. developed a radiomics signature to predict TNM stage of ESCC, resulting in an AUC of 0.762 at internal validation [11]. Unlike our signature that is based on radiomics features extracted from intratumoral and peritumoral areas, Wu et al. extracted and analyzed intratumoral features alone. Our radiomics signature demonstrated a slightly better performance (AUCs: 0.813 vs 0.762), although a head-to-head comparison is needed. One possible explanation was that peritumoral regions may potentially contain complementary predictive information, as aggressiveness is a hallmark of cancer and peritumoral invasion is related to tumor stage [19–22]. Therefore, a combination of intratumoral and peritumoral features, which potentially capture the intratumoral heterogeneity and peritumoral microenvironment simultaneously, could enhance the predictive ability of radiomics in tumor staging in patients with ESCC. Since the developed radiomics signature can reliably predict tumor stage preoperatively, it may contribute to improve selection of ESCC patients most likely to benefit from neoadjuvant therapy while sparing others from the toxic effects of the treatment.

Opposed to previous studies [11, 23, 24] that showed the usefulness of shape-based features (such as tumor volume, tumor length) for assessment of TNM stage, our signature did not include any shape features, likely because shape features do not reflect comprehensively tumor heterogeneity and aggressiveness, thus are less predictive of TNM stage.

Another critical result found in our study was that the radiomics-based staging phenotype was independently associated with RFS, implying its prognostic relevance for ESCC patients. More frequent follow-ups and more positive tailored therapy may be needed for high-risk patients probed by the proposed radiomics signature.

The following study limitations merit consideration. First, the retrospective study may induce inevitable selection bias. Second, we collected patient data from single center. Future multicenter external validation is warranted to validate the generalization of the proposed radiomics signature. Third, manual volumetric segmentation was time-consuming and labor-intensive. Automatic annotation is required to simplify the process in the future. Fourth, we considered the handcrafted radiomic features alone in this study. The role of deep learning features has not been explored. An integrated analysis of the handcrafted and deep learning features may potentially improve predictive performance [25, 26]. Finally, clinical data (such as endoscopic results) for preoperative stage were unavailable in all patients in this retrospective study, so we were unable to compare the efficacy of our radiomics signature with that of clinical stage.

In conclusion, the proposed radiomic signature incorporates features of intratumoral and peritumoral regions, allowing the non-invasive evaluation of tumor stage in ESCC and potentially predicting prognosis.

### Supplementary Information

Below is the link to the electronic supplementary material.

Supplementary file1 (DOCX 14 kb)

Supplementary file2 (PDF 94 kb)

## Data Availability

The data included in the study are available from the corresponding author upon reasonable request.

## Abbreviations

ESCC: Esophageal squamous cell carcinoma
CT: Computed tomography
NLR: Neutrophil-to-lymphocyte ratio
PLR: Platelet-to-lymphocyte ratio
TNM: Tumor-node-metastasis
LN: Lymph node
RFS: Recurrence-free survival
ROI: Region of interest
LASSO: Least absolute shrinkage and selection operator
GLDM: Gray level dependence matrix
GLCM: Gray level co-occurrence matrix
GLRLM: Gray level run length matrix
GLSZM: Gray level size zone matrix
NGTDM: Neighboring gray tone difference matrix
ICC: Intra-class correlation coefficient
AUC: Area under curve
CI: Confidence interval
