# Supplementary material for: Decoding tumor stage by peritumoral and intratumoral radiomics in resectable esophageal squamous cell carcinoma
Source: Abdom Radiol (NY). 2023 Oct 13;49(1):301–11. doi: 10.1007/s00261-023-04061-2 (PMC10789665; doi:10.1007/s00261-023-04061-2)
Supplement: Supplementary file 1 — Supplementary file1 (DOCX 14 kb) [file 261_2023_4061_MOESM1_ESM.docx]

**Supplementary S1**: **CT protocols**

Examinations were performed during a single breath-hold with the patient supine. The scanning coverage was from sternal notch to the middle of the kidneys. The CT parameters were as follows: 120 kVp; 130-280 mAs; 0.4s rotation time; detector collimation: 64 ×0.625 mm; field of view (FOV) 300-400mm; matrix 512 × 512. After routine non-enhanced CT, arterial phase contrast-enhanced CT was started 25–30 s after an intravenous administration of 1.5 ml/kg of the iodinated contrast material (Ultravist 370, Bayer Schering Pharma) at a rate of 3.0 to 3.5 mL/s via a pump injector (Ulrich CT Plus 150, Ulrich Medical). The administration of the contrast medium was followed by a saline flush. The raw data were reconstructed with 5.0-mm section thickness.
