# Supplementary material for: Decoding tumor stage by peritumoral and intratumoral radiomics in resectable esophageal squamous cell carcinoma
Source: Abdom Radiol (NY). 2023 Oct 13;49(1):301–11. doi: 10.1007/s00261-023-04061-2 (PMC10789665; doi:10.1007/s00261-023-04061-2)
Supplement: Supplementary file 2 — Supplementary file2 (PDF 94 kb) [file 261_2023_4061_MOESM2_ESM.pdf]

Image protocol quality – well–documented image protocols (for example, contrast, slice thickness, energy, etc.) and/or usage of public image protocols allow reproducibility/replicability

- [x] protocols well documented
- [x] public protocol used
- [ ] none

Multiple segmentations – possible actions are: segmentation by different physicians/algorithms/software, perturbing segmentations by (random) noise, segmentation at different breathing cycles. Analyse feature robustness to segmentation variabilities

(●) yes

( ) no

Phantom study on all scanners – detect inter–scanner differences and vendor–dependent features. Analyse feature robustness to these sources of variability

( ) yes

(●) no

Imaging at multiple time points – collect images of individuals at additional time points. Analyse feature robustness to temporal variabilities (for example, organ movement, organ expansion/shrinkage)

( ) yes

(●) no

Feature reduction or adjustment for multiple testing – decreases the risk of overfitting. Overfitting is inevitable if the number of features exceeds the number of samples. Consider feature robustness when selecting features

(●) Either measure is implemented

( ) Neither measure is implemented

Multivariable analysis with non radiomics features (for example, EGFR mutation) – is expected to provide a more holistic model. Permits correlating/inferencing between radiomics and non radiomics features

(●) yes

( ) no

Detect and discuss biological correlates – demonstration of phenotypic differences (possibly associated with underlying gene—protein expression patterns) deepens understanding of radiomics and biology

( ) yes

(●) no

Cut–off analyses – determine risk groups by either the median, a previously published cut–off or report a continuous risk variable. Reduces the risk of reporting overly optimistic results

(●) yes

- ○ no

Discrimination statistics – report discrimination statistics (for example, C–statistic, ROC curve, AUC) and their statistical significance (for example, p–values, confidence intervals). One can also apply resampling method (for example, bootstrapping, cross–validation)

- ☑ a discrimination statistic and its statistical significance are reported
- ☑ a resampling method technique is also applied
- ☐ none

Calibration statistics – report calibration statistics (for example, Calibration–in–the–large/slope, calibration plots) and their statistical significance (for example, P–values, confidence intervals). One can also apply resampling method (for example, bootstrapping, cross–validation)

- ☐ a calibration statistic and its statistical significance are reported
- ☐ a resampling method technique is applied
- ☑ none

Prospective study registered in a trial database – provides the highest level of evidence supporting the clinical validity and usefulness of the radiomics biomarker

- ○ yes
- ◉ no

Validation – the validation is performed without retraining and without adaptation of the cut–off value, provides crucial information with regard to credible clinical performance

- ☐ No validation
- ☑ validation is based on a dataset from the same institute
- ☐ validation is based on a dataset from another institute
- ☐ validation is based on two datasets from two distinct institutes
- ☐ the study validates a previously published signature
- ☐ validation is based on three or more datasets from distinct institutes

Comparison to 'gold standard' – assess the extent to which the model agrees with/is superior to the current 'gold standard' method (for example, TNM–staging for survival prediction). This comparison shows the added value of radiomics

- ○ yes
- ◉ no

Potential clinical utility – report on the current and potential application of the model in a clinical setting (for example, decision curve analysis).

- ○ yes
- ◉ no

Cost–effectiveness analysis – report on the cost–effectiveness of the clinical application (for example, QALYs generated)

- ○ yes
- ◉ no

Open science and data – make code and data publicly available. Open science facilitates knowledge transfer and reproducibility of the study

- ☐ scans are open source
- ☐ region of interest segmentations are open source
- ☐ the code is open sourced
- ☐ radiomics features are calculated on a set of representative ROIs and the calculated features and representative ROIs are open source

Total score

12
(33.33%)
